# Enamel Softening Can Be Reduced by Rinsing with a Fluoride Mouthwash Before Dental Erosion but Not with a Calcium Solution

**DOI:** 10.3290/j.ohpd.b2259087

**Published:** 2021-11-05

**Authors:** Philipp Körner, Thanh Phong Nguyen, Blend Hamza, Thomas Attin, Florian J. Wegehaupt

**Affiliations:** a Resident, Clinic of Conservative and Preventive Dentistry, Center of Dental Medicine, University of Zurich, Zurich, Switzerland. Wrote the manuscript.; b Dental Master’s Student, Clinic of Conservative and Preventive Dentistry, Center of Dental Medicine, University of Zurich, Zurich, Switzerland. Performed the experiments in partial fulfilment of requirements for a Master’s degree, proofread the manuscript.; c Resident, Clinic of Orthodontics and Pediatric Dentistry, Center of Dental Medicine, University of Zurich, Zurich, Switzerland. Contributed substantially to discussion, proofread the manuscript.; d Professor and Director, Clinic of Conservative and Preventive Dentistry, Center of Dental Medicine, University of Zurich, Zurich, Switzerland. Research idea, contributed substantially to discussion, proofread the manuscript.; e Head of Division of Preventive Dentistry and Oral Epidemiology, Clinic of Conservative and Preventive Dentistry, Center of Dental Medicine, University of Zurich, Zurich, Switzerland. Research idea, hypothesis, experimental design, contributed substantially to discussion and writing the paper, proofread the manuscript.

**Keywords:** calcium solution, dental erosion, erosive tooth wear, erosion protection, fluoride mouthwash

## Abstract

**Purpose:**

This in-situ-study investigated if rinsing the oral cavity with a calcium containing solution or a fluoride containing mouthwash immediately before an erosive attack leads to reduced enamel softening.

**Materials and Methods:**

Bovine enamel samples (n = 240) with measured baseline surface microhardness (KHN) were assigned to five series (S1–5). Twelve participants carried out each series as follows: Four enamel samples of the associated test series were placed in an intraoral appliance and carried in each participants’ mouth. After 30 min, the participants either rinsed the oral cavity for 60 s with 30 ml of a solution prepared from a 1,000 mg calcium effervescent tablet dissolved in 100 ml water (S2), an 800 mg calcium containing mineral supplement powder (5 g) dissolved in 200 ml water (S3), a fluoride (500 ppm) mouthwash (S4), a fluoride (500 ppm) and stannous chloride (800 ppm) containing mouthwash (S5), or did not rinse with any test solution before the erosive attack (S1, negative control). The participants subsequently rinsed the oral cavity with 100 ml of a soft drink (Sprite Zero) for 60 s to simulate the erosive attack and immediately afterwards with water to stop the erosive process. As final step, surface microhardness was measured a second time and hardness loss (∆KHN) calculated. Differences of ∆KHN between the series were investigated by fitting a mixed effect model to the data set.

**Results:**

The highest loss of microhardness and thus softening of enamel (mean of ∆KHN; lower/upper confidence level) was observed in the negative control (S1: 60.2; 67.6/52.8). While no statistically significant difference (P > 0.05) compared to S1 could be found in S2 (50.0; 57.4/42.5) and S3 (54.6; 62.1/47.2), statistically significantly less softening of enamel (P < 0.001) was discovered in S5 (33.8; 41.2/26.4) and S4 (41.8.2; 49.3/34.4). S5 showed the overall lowest values for ∆KHN and thus best protection from enamel softening.

**Conclusion:**

Rinsing with a fluoride mouthwash or a fluoride and stannous chloride containing mouthwash immediately before an erosive attack reduces the softening of enamel. None of the investigated calcium-containing solutions was able to reduce erosion induced softening of enamel.

Nowadays, there are many ways for people of all ages to face oral acid exposure in their daily life. This might be the reason for the frequently reported and increasing prevalence of dental erosion.^25^ In literature, the term ‘dental erosion’ is used either synonymously to erosive tooth wear or can be described as its primary etiological factor. It can be regarded as the combination of irreversible loss of dental hard tissue and softening of tooth surfaces, caused by demineralising, acidic agents without the influence of microorganisms.^32^ The acidic agents are generally known to be either from intrinsic^8^ or extrinsic^23^ origin. Extrinsic acids are brought into the oral cavity from the outside and get in contact with teeth during the consumption of acid containing beverages,^24^ foodstuff,^23^ or medicaments.^16^ Intrinsic acid occur in gastric fluid mainly composed of hydrochloric acid^18^ and get into the oral cavity during vomiting^31^ or reflux.^9^ In advanced stages, the loss of dental hard tissues may lead to functional and aesthetic impairments^2^ but also to pain due to exposed dentine surfaces or even pulp exposure.^12^

Generally, dental erosion takes place if teeth are brought into contact with a solution that is undersaturated with respect to tooth minerals. The main factors responsible for the degree of saturation are the concentration of essential tooth minerals (calcium/phosphate) within the solution and the presence of acids (pH value). In case that the concentration of essential tooth minerals surrounding a tooth is within the range of physiological saliva, but the pH value falls below the respective critical values^19^ (enamel: pH 5–5.5; dentine: pH 6–6.5), erosive demineralisation and softening of dental hard tissues occurs. However, it has been shown that attacks below the critical pH values can be tolerated by teeth without damage if the attacking solution is oversaturated in tooth minerals, ie, if the concentration of minerals, especially calcium,^3,4,36^ in the acidic solution is higher than in physiological saliva. It might therefore be speculated that the erosive softening of dental hard tissues could also be reduced if a higher content of tooth minerals was surrounding the teeth during an erosive attack. This might be implemented by rinsing the oral cavity with a mineral (especially calcium) containing solution immediately before an erosive attack. The temporarily increased amount of minerals might function as a reservoir providing minerals to the acid and lead to an oversaturated intraoral milieu. This way, a loss of tooth minerals and the demineralisation of dental tissue during the erosive attack could possibly be reduced or even prevented. Based on this consideration, a recent study^20^ investigated whether rinsing the oral cavity with different calcium containing solutions prior to an erosive attack reduces the softening of enamel. It was speculated that especially in patients with intrinsic dental erosion caused by deliberately induced vomiting (bulimia nervosa, anorexia nervosa) this approach might attenuate the softening of enamel. The results indicated that none of the investigated calcium-containing solutions was able to significantly reduce erosion-associated softening of enamel. Due to an obvious tendency towards an increased protective effect with increased calcium concentration, it was nonetheless considered worthy following up with even higher concentrations. Besides, statistically significant reduction of enamel softening was observed in the study^20^ by rinsing the oral cavity with a fluoride (500 ppm) and stannous chloride containing mouthwash before an erosive attack. It could however not be conclusively clarified whether the protective effect could mainly be attributed either to solely the fluorides or rather the combination of fluorides and stannous chloride contained in the tested mouthwash.

Therefore, the aim of the present study was to evaluate if rinsing the oral cavity with highly concentrated calcium solutions or fluoride-containing mouthwashes (with and without stannous chloride) immediately before an erosive attack reduces softening of enamel and thus enables protection from the erosively induced alteration of dental hard tissue.

## Materials and Methods

### Sample Preparation

Tooth collection was carried out in accordance with relevant guidelines and regulations and did not require additional approval. Extracted bovine incisors were used to gain a total of 300 enamel samples. Enamel cylinders with 3 mm diameter were cut from the incisors’ buccal surfaces using a water-cooled diamond trephine mill (BFW 40/E, Proxxon; Föhren, Germany) and afterwards embedded in acrylic resin (Paladur, Heraeus Kulzer; Hanau, Germany). Enamel surfaces were ground flat and polished in an automatic grinding machine with a pressure of 5 N and 150 rpm (Tegramin 30, Struers; Birmensdorf, Switzerland) using water-cooled carborundum discs (Waterproof Silicon Carbide Paper, Struers) with decreasing grain size (1,200 grit, 5 s; 2,000 grit, 20 s; 4,000 grit, 40 s). The baseline surface microhardness (KHN) of all 300 produced samples was measured subsequently. From these 300 enamel samples, a total of 240 with comparable measured microhardness were stratified and allocated to five experimental series. The mean KHN value of each series was 333, within the series the KHN values reached from 295 to 373 (± 12% deviation from mean KHN). Finally, all allocated samples were exposed to gamma sterilisation (12 kGy, 4 h, Paul Scherrer Institut; Villigen, Switzerland) and stored in tap water until the respective experimental series was performed.

### Study Participants

A total of 12 volunteers (6 female and 6 male) participated in this in-situ-study. The exclusion criteria were as follows: under 18 years of age; non-compliance; current orthodontic treatment hampering the insertion of an intraoral appliance; contemporaneous participation in another clinical study; alcohol or drug abuse; heavy smoking (> 10 cigarettes/day); patients with known allergies against products to be used in the study or patients with hyposalivation (unstimulated: < 0.25 ml/min in 15 min; stimulated: < 1.0 ml/min in 5 min).^11^ Each participant gave written informed consent. The study was approved by the Swiss Ethics Committees on research involving humans (project ID 2018-02141).

### Study Design

**Fig 1:**
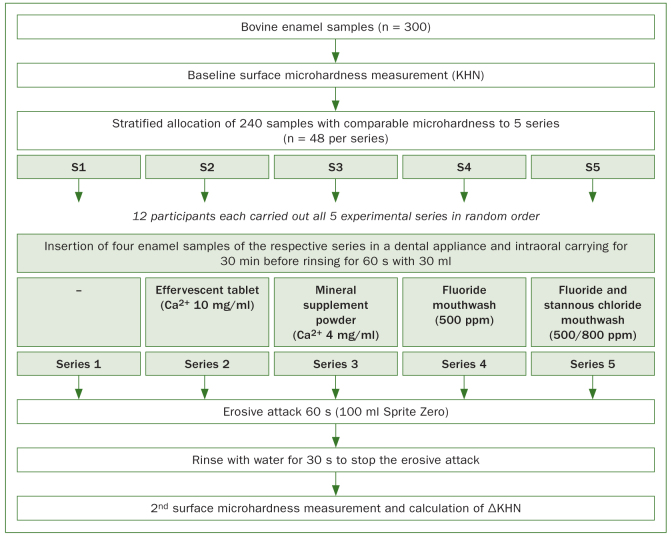
Experimental design.

The experimental design is illustrated in Figure 1. In five experimental series (S1–5), four different calcium or fluoride containing test solutions were investigated for their potential to reduce enamel softening in the course of dental erosion. Except from the negative control (S1) where no solution was applied before the erosive attack, one solution was applied in each series (S2–4). The participants received an individually fabricated, custom made intraoral appliance, able to carry four enamel samples in the area buccal of the left and right maxillary second premolar and first molar. Each participant performed all five experimental series. A wash-out period of 7 days separated the respective experimental series. The sequence was randomly assigned for each participant. Seven days before the first experimental series, the participants started using a commercial fluoride-containing toothpaste (Elmex Caries Protection, GABA; Therwil, Switzerland) and were instructed not to eat or drink 2 h before and during the experiments.

At the beginning of an experimental series, four enamel samples of the associated test series were inserted in the intraoral appliance and carried in each participants’ mouth to adapt to the individual intraoral conditions and enable pellicle acquisition which was shown to adhere rapidly to enamel and have a modifying and protective effect on dental erosion.^15^ After 30 min, the participants either rinsed the oral cavity with 30 ml of a solution prepared from a 1,000 mg calcium (4,954 mg Calcium-D-gluconat – Calciumlactat (2:3) 2H_2_O and 900 mg Calciumcarbonat) effervescent tablet (Calcium-Sandoz Fortissimum 1,000 mg, Hexal; Holzkirchen, Germany) dissolved in 100 ml water (⩠ Ca^2+^ 10 mg/ml) (S2), 30 ml of a solution prepared from a 800 mg calcium (Calciumcarbonat)-containing mineral supplement powder (Probase powder, Burgerstein; Rapperswil-Jona, Switzerland), 5 g dissolved in 200 ml water (⩠ Ca^2+^ 4 mg/ml) (S3), with 30 ml of a fluoride-containing mouthwash (CB12, 500 ppm NaF, MEDA Pharma; Wangen-Bruettisellen, Switzerland) (S4), 30 ml of a fluoride and stannous chloride-containing mouthwash (Elmex protection erosion, 375 ppm NaF, 125 ppm, 800 ppm Sn^2+^, GABA) (S5), for 1 min, or did not rinse with any test solution before the erosive attack (S1, negative control). Immediately (within 15 s) after finishing this step, the participants consecutively rinsed their oral cavity two times for 30 s with 50 ml of a commercial soft drink at room temperature (Sprite Zero, pH 3.14, Coca Cola Schweiz; Bruettisellen, Switzerland) to simulate an erosive attack (total erosive time = 60 s). Subsequently, the participants instantly rinsed their mouth for 30 s with tap water to dilute and neutralise the acid and stop the erosive processes. Afterwards, the four samples were carefully removed from the appliances and the surface microhardness was measured a second time. The acid-induced loss of microhardness within this experimental design was tested in an internal pilot investigation and verified in a recent study with similar experimental set up.^20^ Compared to the last mentioned study, the same kind of effervescence tablet but with double the amount of calcium (1,000 mg instead of 500 mg) was used in S2. The 800 mg calcium-containing base powder in S3 advertises acid-base regulation and showed a protective effect on erosive enamel wear in another study.^36^ Because of more or less noticeable differences in taste, colour and consistency of the test solutions, the study could not reliably be performed blinded for participants and investigator, but at least was blinded during microhardness measurement. The investigated calcium- and fluoride-containing products are commercially available and approved for oral application. Further information and details about the active ingredients of the different products (S2–S5) are given in Table 1.

**Table 1:** Information and details about the active ingredients of the different products used in the study

Calcium products	Series	Active ingredients	Manufacturer
Calcium-Sandoz Fortissimum 1,000 mg	S2	1 effervescence tablet contains: 1,000 mg (25 mmol) Calcium (= 4,954 mg Calcium-D-gluconat – Calciumlactat (2:3) 2H_2_O and 900 mg Calciumcarbonat)	Hexal; Holzkirchen, Germany
Probase powder	S3	5 g powder contain: Calciumcarbonat (800 mg), Potassiumbicarbonat (575 mg), Magnesiumcarbonat (300 mg), Zincgluconat (10 mg), Mangangluconat (2 mg)	Burgerstein; Rapperswil-Jona, Switzerland

Fluoride mouthwashes	Series	Active ingredients	Manufacturer
CB12	S4	Sodiumfluorid (500 ppm) (Zinc acetate dihydrate 0.3%) (Chlorhexidine diacetate 0.025%)	MEDA Pharma; Wangen-Bruettisellen, Switzerland
Elmex protection erosion	S5	Sodiumfluorid (375 ppm) Amine fluoride (125 ppm) Stannous chloride (800 ppm from SNCl2)	GABA; Therwil, Switzerland

### Surface Microhardness Measurement

Surface microhardness (KHN) was measured for a first time before stratified allocation (baseline) and for a second time after each respective experimental series (final). Therefore, the enamel surface of each sample was indented five times (load weight 50 g, indentation time 20 s) using a Knoop hardness-measuring device (High Quality Hardness Tester, Buehler; Duesseldorf, Germany). The distance between the indentations was set to a minimum of 50 µm to each other. Microhardness measurement was performed by a blinded co-worker. After calculating the mean surface microhardness per sample, the loss of surface hardness (∆KHN), as indicator for enamel softening and demineralisation, was determined by subtracting the final microhardness from the baseline microhardness of the respective sample. A low mean value for ∆KHN correlates with a low degree of enamel softening and thus better protection of the respective solution from erosion induced softening.

### Statistical Analysis

Based on a repeated measures design using the same subjects, a mixed-linear model was fitted to the data with ∆KHN as target variable, series as explanatory variable, initial hardness as co-variable (to adjust for differences in the initial hardness of the samples) and participant as random effect. After thoroughly checking the model assumptions, marginal means of ∆KHN per series were calculated and compared pairwise (P-value for multiple comparisons was adjusted according to Tukey). The level of statistical significance was set at 5%. The entire statistical analyses and plots were performed using the statistical software R^29^ including the packages lmerTest^21^ and emmeans.^22^

## Results

**Fig 2:**
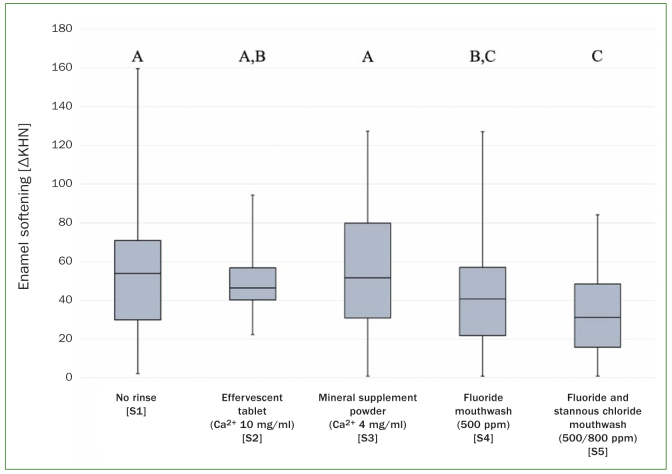
Boxplot of enamel softening (∆KHN) for the different series (S1–5). The lower the softening, the better the respective products prevent the erosion-induced softening. The horizontal line in the box represents the median value, the box represents the 25th and 75th percentile and whiskers represent the 5th and 95th percentile. Not statistically significantly different values are marked with same capital letters.

Enamel softening (∆KHN) in the course of dental erosion and the comparison between the different test series are illustrated in Figure 2 and Table 2. The highest loss of microhardness and thus softening of enamel (mean of ∆KHN; lower confidence level/upper confidence level) was observed in the negative control (S1: 60.2; 67.6/52.8), where no fluoride- or calcium-containing solution was applied before the erosive attack. While no statistically significant difference (P > 0.05) compared to the negative control (S1) could be found in the series where the oral cavity was rinsed with a 1,000 mg calcium solution (S2: 50.0; 57.4/ 42.5) or a 800 mg calcium-containing mineral supplement solution (S3: 54.6; 62.1/47.2), statistically significantly less softening of enamel (P < 0.001) was discovered in the series in which the oral cavity was rinsed with a fluoride containing (S4: 41.8; 49.3/34.4) or fluoride and stannous chloride-containing mouthwash (S5: 33.8; 41.2/26.4). The last-mentioned solution (S5) showed the overall lowest values for ∆KHN and thus best protection from enamel softening.

**Table 2:** Mean values of enamel softening ΔKHN and lower/upper confidence level in the different series (S1–5)

	Series	ΔKHN (mean)	Lower confidence level	Upper confidence level
No rinse	S1	60.2	67.6	52.8
Effervescent tablet (Ca^2+^ 10 mg/ml)	S2	50.0	57.4	42.5
Mineral supplement powder (Ca^2+^ 4 mg/ml)	S3	54.6	62.1	47.2
Fluoride mouthwash (500 ppm)	S4	41.8	49.3	34.4
Fluoride and stannous chloride mouthwash (500/800 ppm)	S5	33.8	41.2	26.4

## Discussion

The results of the present study indicate that rinsing the oral cavity with a 500 ppm fluoride-containing mouthwash (with and without stannous chloride) immediately before an erosive attack reduces the acid induced softening of enamel statistically significantly. However, no statistically significant protective effect is provided in case that high concentrated calcium solutions are applied.

Enamel specimens used in this study were prepared from bovine incisors, which have been used and discussed in multiple studies investigating erosive softening of dental hard tissues and are generally regarded as a suitable substitute for human enamel.^5^ Changes in surface hardness of erosively altered dental hard tissues are commonly assessed using microhardness measurement.^6^ This technique enables reliable quantification of surface softening and the associated dissolution of enamel without causing damage to the sample surface and thus allowing repetitive measurements.

In the introduction section was hypothesised that especially for patients with intrinsic dental erosion caused by deliberately induced vomiting, rinsing with a calcium or fluoride containing solution might attenuate the softening of enamel. Hydrochloric acid (HCl) is a main component of gastric fluid. However, the erosive attacks in this study were simulated using an extrinsic acid (citric acid) in form of a commercial soft drink (Sprite Zero, pH 3.14),^30^ which has to be attributed to ethical concerns. Nevertheless, the utilised soft drink was shown to have a considerable erosive potential,^10, 27, 34^ which is similar to gastric fluid. In literature, the mean pH value of gastric acid is specified at 2.92^8^ and about 3.8 (average pH of gastric contents of bulimic patients).^26^ Other studies describe citric acid as equally or even more erosive than HCl.^7, 14^ Given that a solution provides protection from erosive softening under severe conditions (citric acid), it can be assumed that this effect is also exhibited under less severe conditions (hydrochloric acid). The applied total duration of 60 s for each erosive attack aimed to simulate realistic conditions as they might occur during the consumption of a soft drink or contact of gastric fluids with teeth during vomiting. Dilution and dissociation effects of the acid were counteracted by refreshing the attacking acid (soft drink) after 30 s. However, it has to be pointed out that in case of gastro-oesophageal reflux disease the exposure time might be extended during night-time, which has not been taken into account in this study.

Further limitations might be seen in more or less noticeable differences in taste, colour and consistency of the solutions, wherefore the study could not reliably be performed blinded for participants and investigator, but at least was blinded during microhardness measurement to avoid bias of the results. The calcium content of saliva and/or pellicle and further parameter of the test solutions, such as substantivity in the oral cavity, dissolution of calcium, viscosity or the presence of proteolytic enzymes were not assessed in this study, but still might have an influence on the outcome. However, the focus of this study was to investigate the potential of the tested solutions in combination with the applied technique to reduce enamel softening. In case that a protective effect is found for a solution, the parameters mentioned, including their interaction, would be of high interest to be investigated in a next step.

The results show a statistically significant reduction of enamel softening for the fluoride-containing series. However, no statistically significant protective effect was observed for both series in which a high concentrated calcium solution was applied. Prior to the study it was hypothesised that an increased calcium concentration in the oral cavity during an erosive attack may lead to reduced dental erosion due to an oversaturated intraoral milieu and thus increased saturation of the acid.^37^ The promising tendency reported in a recent study^20^ (similar experimental setup) towards a protective effect with increased calcium concentration could nonetheless not be confirmed in this study although double the amount of calcium (1,000 mg instead of 500 mg in 100 ml water) was used in S2. It might be speculated whether the obtained reservoir of calcium in the oral cavity providing minerals to the attacking acid was still too low to reduce dental erosion, if there was not enough time for interaction between acid and minerals or if the saturation of the acid in the close surrounding of the enamel surface was insufficient. The findings might rather not be explained by a lack of solubility of Ca^2+^ as the examined calcium effervescent tablet contains the two calcium salts calcium lactate gluconate and calcium carbonate which readily dissolve in water to make the active, ionised form of Ca^2+^ freely usable. It has to be considered though that calcium carbonate is hardly soluble in water, which is why citric acid is contained in the effervescent tablet to substantially increase the solubility and release Ca^2+^ from the calcium salt. Whereas, in the mineral supplement solution in S3 only calcium carbonate without any acidic component or easily soluble calcium salts are contained so that it might be speculated that there was only a minor amount of freely usable ionised Ca^2+^ in the oral cavity during the erosive attack. Besides, the results indicate that other minerals in this solution (magnesium, potassium) and trace elements (zinc, manganese) are also not able to provide a protective effect on enamel softening. A further consideration might be that other than the fluoride-containing solutions, the calcium solutions likely did not decisively interact with or modify the enamel surface and did not enable the formation of a protective layer thus making it less resistant to mineral dissolution. These properties might be the reason for the statistically significant reduction of enamel softening in case that the oral cavity was rinsed with a fluoride-containing mouthwash before the erosive attack. Different studies showed that an application of fluorides before an erosive attack can reduce erosive loss of dental hard tissues.^17, 20, 35^ The protective effect of fluoride might be explained by the formation of a CaF_2_-layer on the enamel surface which can be imagined as a resistant and protective surface coating against the attacking acid. The layer functions as a mechanical barrier and also provides a reservoir of minerals able to buffer or deplete hydrogen ions from the acid.^38^ Additionally, fluoride is released from the CaF_2_-layer during an acidic attack and can be incorporated into tooth mineral by forming fluorapatite or fluorohydroxyapatite with decreased susceptibility to further dissolution.^38^

Both fluoride-containing mouthwashes in this study are commercially available over-the-counter products and contain the same total amount of fluoride (500 ppm). However, there are differences in the fluoride compounds. While the fluoride mouthwash in S4 contains only NaF (500 ppm), the fluoride and stannous chloride-containing mouthwash in S5 contains NaF (375 ppm) and AmF (125 ppm). It was shown that, at the same concentrations, AmF may be more effective than NaF to protect enamel from acid.^38^ Besides, the stannous ion (800 ppm Sn_2_+ from SnCl_2_) contained in S5 is known to be a potent reactant with hydroxyapatite and may further reduce the solubility of dental hard tissue.^33^ Hence, the two fluoride compounds (NaF and AmF) and stannous chloride might have interacted with the outer enamel, reducing its solubility and building a glaze layer of Sn_2_OHPO_4_, Sn_3_F_3_PO_4_, Ca(SnF_3_)2 and CaF_2_, thus providing a resistant and protective surface coating against the attacking acid.^39^ Accordingly, the overall highest surface protection in this study was observed for the fluoride and stannous chloride containing mouthwash (S5). Numerous other studies describe equally pronounced and effective anti-erosive properties for this product.^1, 13, 28, 39^ Therefore, the first choice in terms of reducing enamel softening in the course of dental erosion should be rinsing with a fluoride and stannous chloride-containing mouthwash before the erosive attack, but still, an only fluoride (500 ppm)-containing mouthwash can also be recommended.

It should be kept in mind though that the most effective way to prevent dental erosion is the reduction of the frequency of tooth contact with dental erosion-causing acids. For patients with signs of erosion caused by deliberately induced vomiting as manifestation of psychosocial disorder, it might however be futile to stop the pathogenesis. For these patients, rinsing the oral cavity with a fluoride or preferably with a fluoride and stannous-chloride-containing mouthwash immediately prior to the self-induced vomiting might reduce tooth damage and can be recommended.

## Conclusion

Within the limitations of the present study, it can be concluded for the investigated products that rinsing with a fluoride mouthwash or a fluoride- and stannous-chloride-containing mouthwash immediately before an erosive attack can reduce but not totally hamper the erosion-induced softening of enamel. None of the investigated high concentrated calcium-containing solutions is able to statistically significantly reduce the erosion-induced softening of enamel.
